# Composite Lymphoma’s Ability to Adapt and Change Through Plasticity: A Case Report and Literature Review

**DOI:** 10.7759/cureus.45696

**Published:** 2023-09-21

**Authors:** Haneen Al-Maghrabi, Jaudah Al-Maghrabi

**Affiliations:** 1 Department of Pathology, King Faisal Specialist Hospital and Research Center, Jeddah, SAU; 2 Department of Pathology, King Abdulaziz University Faculty of Medicine, Jeddah, SAU

**Keywords:** diffuse large b lymphoma, epstein barr virus (ebv), classical hodgkin lymphoma, composite lymphoma, synchronous

## Abstract

Composite/synchronous lymphoma is an uncommon condition that presents a challenge in histopathological diagnosis due to the simultaneous appearance of two or more lymphomas, including instances where they are intermixed within the same location. Performing a core needle tissue biopsy adds a challenge for pathologists when trying to diagnose a condition that requires excisional tissue for a thorough evaluation. This report highlights a distinctive instance of composite lymphoma in which classical Hodgkin lymphoma (cHL) is combined with diffuse large B-cell lymphoma (DLBCL). All pertinent information including clinical, histopathological, and immunohistochemical data for each of these composite lymphomas is provided. In addition, we conducted a literature review of the published data. The findings from these data further support the theory of a shared clonal origin and transdifferentiation occurrence in the process of lymphoma development.

## Introduction

The presence of a minimum of two distinct pathological types of lymphomas in the same anatomical location characterizes a composite lymphoma [1]. Composite/synchronous lymphomas, as defined by Custer et al. in 1954 [2], are also considered to have occurred when multiple lymphomas manifest in different locations simultaneously [3]. Both synchronous and composite lymphomas may have a common pathophysiology/ pathogenic gene due to the identification of shared cellular origins between the two pathologies. The fact that various types of lymphomas occur at the same time makes them ideal examples for investigating the adaptability of cells in the development of lymphomas. Since the beginning of the 21st century, composite lymphomas that are composed of classical Hodgkin lymphoma (cHL) have sparked significant pathology interest. Initially, the simultaneous presence of cHL and non-Hodgkin lymphomas, which exhibit disparate morphological pathologies, was assumed to be a chance of coincidental association. However, with the use of molecular and genetic studies in the recent decade it has been discovered that there are common molecular abnormalities shared between these two types of lymphomas [3]. In addition, research has found that there are commonly occurring genetic translocations between the composite lymphomas involving BCL2, BCL6, and CCND1 (Cyclin-D1) [4]. Chain gene rearrangements of IgH that encode the heavy chain and/or IgK gene encode light chains of immunoglobulins were also detected. There were also identified common pathogenic genes that existed between both groups such as TP53, BCL2, ARID1A, BCOR, KMT2D, EP300, and SF3B1 [4-6]. Thus far, cHL has been observed in combination with nodular lymphocyte-predominant Hodgkin lymphoma (NLPHL), as well as various types of B-cell lymphomas most commonly follicular lymphoma (FL), and diffuse large B-cell lymphoma (DLBCL), others such as mantle cell lymphoma (MCL), marginal zone lymphoma (MZL), in addition to T-cell lymphomas [7]. Interestingly, it seems that cHL/B-cell composite lymphomas exhibit a less aggressive clinical course compared to the clinical outcome of cHL/B-cell sequential lymphomas, which are characterized by the development of multiple lymphomas in a patient, one after the other. However, the differentiation between composite and sequential lymphomas may not always be clear in clinical and pathological settings. The available data in the literature on composite lymphomas relies solely on individual cases and limited series studies, which hinder a comprehensive analysis of their clinical, histopathological, molecular, and pathophysiological aspects. To expand our comprehension of composite lymphoma adaptability, we report an interesting case of a submandibular lymph node with two different pathology interpretations on needle biopsy, that turned out unexpectedly composite lymphoma on excisional biopsy.

## Case presentation

**Figure 1:**
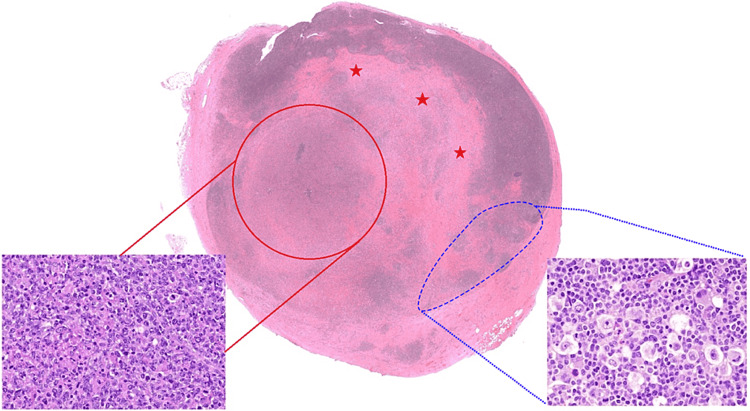
Representative histology of wholemount section tissue after excisional lymph nodal biopsy which includes areas of diffuse large B-cell lymphoma (red circle and inset), classical Hodgkin lymphoma (blue circle and inset), (hematoxylin & eosin (H&E); insets original magnification ×200). The presence of a composite pathology in a single lymph node accounts for the distinct pathology results obtained from the fine needle biopsies taken from two different areas - the blue area indicating the site of the first biopsy, and the red circle denoting the location of the second biopsy. Note the thick fibrous separating bands (red stars). This highlights the significance of excisional biopsy for accurate pathology diagnosis.

**Figure 2:**
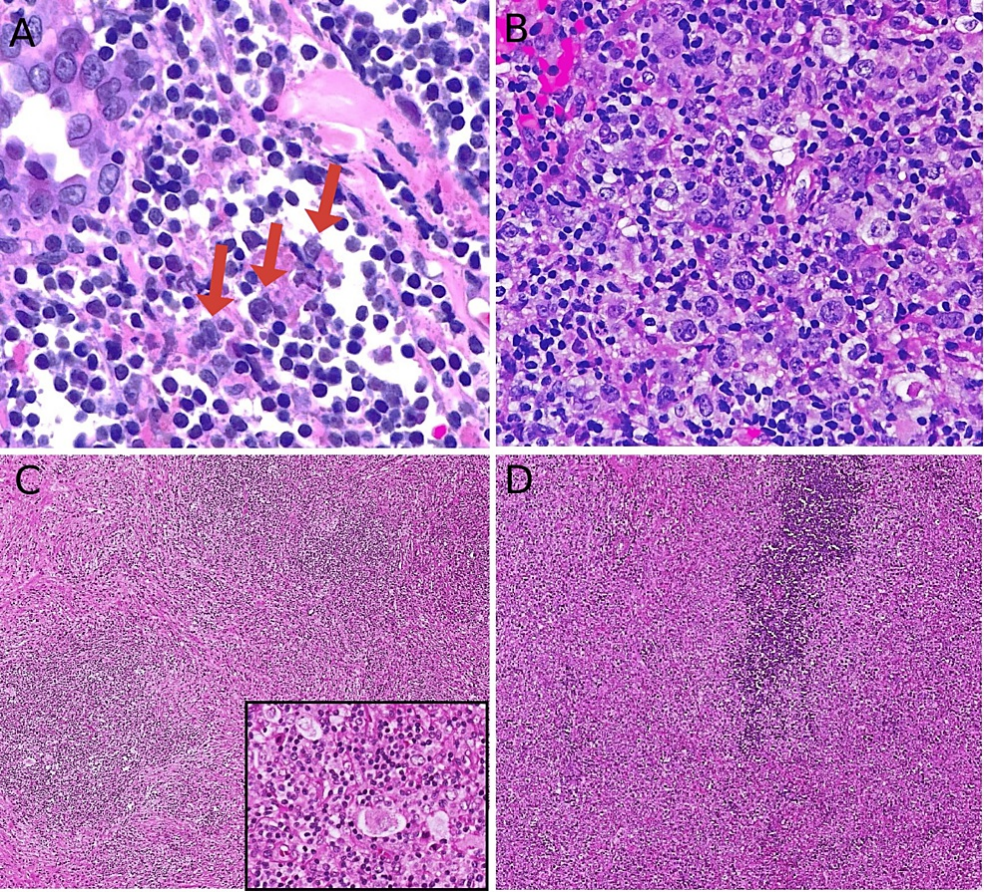
Histopathology examination by hematoxylin and eosin stain (H&E). (A) First biopsy taken from intraparotid lymph node that show mostly necrosis, foci reveal large binucleated atypical cells (red arrows) compatible with classical Hodgkin's lymphoma, note the small reactive lymphocytes in the background (H&E; 40x); (B) second biopsy taken from the same location reveals sheets of large, atypical B-cells that were consistent with diffuse large B-cell lymphoma (H&E; 40x); (C) tissue resection after excisional lymph node sampling showing area of nodular lymphocytic proliferation, eosinophils in background, with prominent RS-cells in between (inset H&E; 40x); (D) adjacent area show diffuse sheets of large atypical cells, prominent nucleoli, and frequent mitosis seen consistent with diffuse large B-cell lymphoma (H&E; 40x).

**Figure 3:**
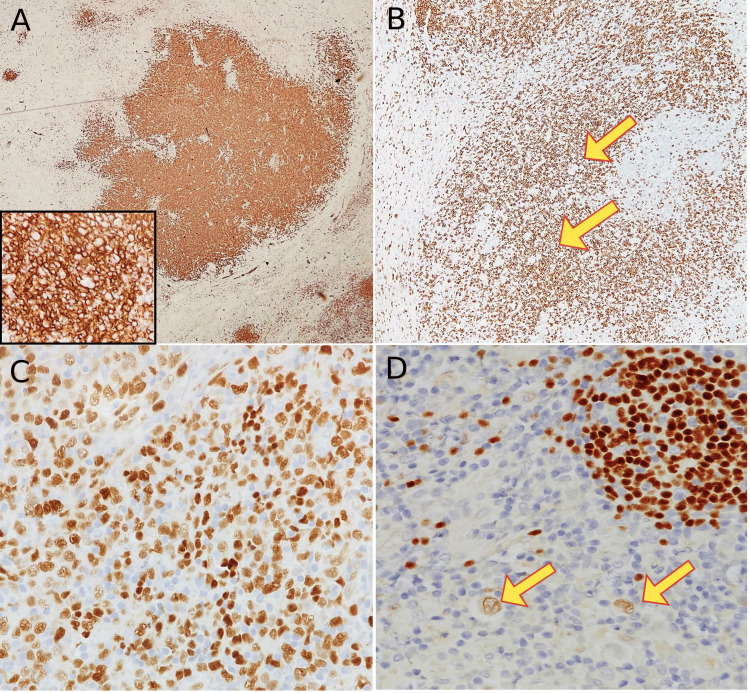
Immunohistochemistry studies done on the excisional lymph node diagnosed with composite classical Hodgkin lymphoma and diffuse large B-cell lymphoma. (A) CD20 immunostaining highlights strong expression in diffuse sheets of large, atypical cells; a component of diffuse large B-cell lymphoma (4x), high power magnification (40x) (inset); (B) CD20 in the adjacent area were negative in RS-cells (yellow arrow), note the positive background of small B-lymphocytes (4x); (C) PAX-5 shows strong nuclear staining in diffuse large B-cell lymphoma component (20x); (D) PAX-5 shows dim nuclear staining in RS cells confirming the classical Hodgkin lymphoma component (yellow arrow) (20x).

This is a healthy 45-year-old gentleman, presented with left parotid gland swelling, otherwise healthy, non-smoker, negative past medical history. He underwent a superficial parotidectomy of the left side at an outside facility and was referred to our hospital to rule out lymphoma. After a comprehensive physical examination and various medical laboratory tests, axial contrast-enhanced computerized tomography (CT) scan of the neck soft tissue was performed with coronal and sagittal reconstruction. It showed a well-defined homogeneously enhancing lesion, which could be related to an enlarged intraparotid lymph node. No manifestation of intrathoracic and intra-abdominal lymphoma was seen. The large, atypical cells are diffusely positive for CD20, PAX-5, and CD45. Ki-67 positive in 70% of atypical cells. The pathologist recommended excisional biopsy /parotidectomy for better and more accurate pathological assessment. Given the two distinct and perplexing outcomes of the initial biopsies, the team opted to perform an excisional biopsy on the affected lymph node. Indeed, we received a left submandibular lymph node excisional biopsy that revealed two district components (Figure 1), one of a nodular growth pattern within a thickened lymph nodular capsule, replacing the lymph node. The team requested to review outside pathology slides material which was done on excisional biopsy of left superficial parotidectomy that showed atypical lymphoid infiltrate with extensive necrosis suspicious for classical Hodgkin's lymphoma (Figure 2A). Only residual lymphoid infiltrates at the periphery were seen that show atypical large cells negative for CD45, and positive for CD30 and CD15. Epstein-Barr encoding region (EBER) in situ hybridization (ISH) was negative at the submitted material. Unfortunately, due to the nature of the specimen and the presence of atypical lymphoid infiltrate at the periphery of the tissue other markers were not of a big help in that case. The medical team in charge sent another left parotid lymph node biopsy. Which reveals atypical large B-cell lymphocytic proliferation (Figure 2B). A varying number of Reed-Sternberg cells (RS cells), small lymphocytes, and eosinophils are present (Figure 2C). These RS cells are positive for CD30, CD15, PAX-5 (dim), and MUM-1. Ki-67 proliferative index is low (around 20%). Additionally, there is another component that has a diffuse pattern consisting of a large proliferation of atypical B-cells (Figure 2D). Mitotic figures that are easily identifiable are present, indicating DLBCL. These sheets of cells are positive for CD45, CD20, PAX-5 (strong), CD10, BCL-6, and BCL-2. Ki-67 proliferative index in that area is 80% (Figures 3A-3D). Based on the above histologic findings and the interpretation of immunohistochemistry results, it is believed that the patient has a composite lymphoma consisting of cHL (NS-subtype) and DLBCL (germinal center B-cell subtype). The case was discussed at the multidisciplinary lymphoma tumor board and staged IE, decided for chemotherapy, and reassessed for consolidation by involved filed radiation therapy (IFRT) according to the patient`s disease response. The patient received three cycles of chemotherapy CHOP-R; the last one was six months ago. Whole body fluorodeoxyglucose (FDG)-positron emission tomography (PET) scan was performed as per department standard protocol. Comparison is made of prior FDG PET-CT scan from last year, which showed Significant interval metabolic and morphologic improvement of the previously noted left upper cervical lymphadenopathy, indicating excellent response to therapy. No new concerning size significant FDG avid nodal or extranodal disease. No evidence of focal FDG avid brain lesion. The patient is alive, healthy, and happily living with his family up until the day of the case report.

## Discussion

It is uncommon to encounter composite/synchronous lymphoma, which is challenging to diagnose histopathologically, especially with tissue limitations such as core needle biopsy. Because of the rarity of these diseases, occurring in less than 0.5% of lymphomas, it is difficult to conduct prospective studies, making data limited to case reports and small series. Reviews showcase that over 70% of patients who have composite lymphomas are aged 55 or older, which indicates that advanced age and host immune status could be potential factors that increase the risk of developing synchronous lymphomas [8]. Nonetheless, instances of cHL/NLPHL [9], cHL/DLBCL [10], and cHL/T-cell lymphoma [11] in individuals below the age of 35 have also been documented, attesting to the fact that this pathogenesis can arise at any stage of life. Histopathology analysis of published data often reveals that the cHL group predominantly consists of mixed cellularity (MC-type), which could imply a specific underlying pathophysiology linked to these subtypes. Furthermore, composite lymphomas frequently exhibit focal and/or weak expression of CD20 in Hodgkin cells ranging from 28% to 55%, whereas isolated de novo cHL cases report less frequent expression, with a range of 12%-28% [12]. Moreover, the Hodgkin cells may display markers that are linked to the lymphoma they are associated with, such as BCL2 and BCL6 in FL and Cyclin-D1 in MCL, as well as similar gene rearrangement in both contingents. Further supporting the notion that there is a shared clonal ancestry. The current basis for non-cHL, B-cell classification of lymphomas, depends on the differentiation between germinal center and post-germinal center subgroups and lies in cellular origin. We believe that the level of cellular plasticity during composite lymphomagenesis seems to be higher than initially believed. This cellular plasticity is well exemplified by FL, which arises from germinal centers and has been known to convert into post-germinal center DLBCL or transdifferentiate into myeloid diseases like histiocytic/dendritic cell sarcomas in rare occurrences [13].

When diagnosing, the difficulty lies in distinguishing a group of cHL from Hodgkin-like cells that may be present in B-cell or T-cell lymphomas. A core needle submission for diagnosis may prove to be more difficult or impossible to diagnose accurately, as the submitted material may not adequately reflect the pathology of the combined lymphoma target. This could result in an incomplete diagnosis. Therefore, it is always recommended to use a complete excisional biopsy for a more accurate pathology diagnosis. Distinguishing cHL from Hodgkin-like/ or RS-like cells can be a challenging task for pathologists. To identify Hodgkin-like cells, certain criteria need to be taken into consideration; these include the absence of fibrosis and/or cHL stromal reaction cells as eosinophils and the presence of histiocytes. Mixed/non-nodular distribution of Hodgkin cells, with weak expression of common cHL markers as CD30 and CD15, along with intense (strong) expression of CD45 and B-cell markers - with bright PAX-5 and CD20, unlike the classical RS cells in cHL with express dim/weak nuclear PAX-5 and mostly negative CD20 and CD45. Additionally, the existence of reactive germinal centers or post-germinal center immunoblasts proliferation with a regular differentiation pattern does not indicate a diagnosis of cHL. The difficulty in achieving the distinction between different differential can often arise in practice, particularly when dealing with EBV lymphoproliferative disorders. In such cases, investigating the latency of EBV may prove to be beneficial [14].

In cases of CHL and follicular composite lymphomas most patients who present do not have a previous history of lymphoma and are typically middle-aged or older. The majority of the cHL group belongs to the MC-type, while a smaller portion belongs to the nodular sclerosis (NS-type). The FL component is typically a distinct group that predominantly consists of low grade (typically grade 1 and 2). Studies suggest that the group of cHL within the cHL/FL composite could be distinct from standalone cHL. In a study, patients who received chemotherapy for a composite lymphoma-like condition had a slightly lower rate of relapse or death compared to other patients with cHL alone, emphasizing the need to treat both contingents [4]. According to a study, a morphological contrast has been observed, with a greater occurrence of EBER-negative cHL-MC subtype as compared to the EBER-positive denovo cHL-MC, suggesting that EBV infection may not be as common in composite lymphoma cases. Furthermore, immunohistochemical variations have been observed, such as a higher rate of immunohistochemistry expression of centrofollicular differentiation markers and certain B-cell markers in cHL, which raises doubts regarding the efficacy of anti-CD20 antibody therapy for the cHL counterpart [15]. There are only a few cases of cHL/MCL composite lymphomas reported in the literature, making it a less common occurrence than cHL/FL composite lymphomas. The typical clinical presentation involves older males who have a previous medical history of MCL. They usually present with multiple adenopathy but do not exhibit B symptoms. Frequent positivity of Hodgkin cells for CD15 and EBER is commonly observed in both groups. There is data accessible that suggests that a transdifferentiation of cHL may arise from a tumor mantle cell precursor, and it is possible that the cHL contingent could go through the germinal center [6]. Composite lymphomas involving cHL and MZL are typically observed in elderly males who do not have a prior history of lymphoma. These patients present with adenopathy but without the presence of B symptoms. Expression of p53 and other markers such as CD30, CD15, LMP-1, and EBER, which are typically observed in the CHL-MC type. However, the MZL contingent does not exhibit these markers. There was a slight decrease in the rate of disease relapse and death among patients who received chemotherapy for small/diffuse B-cell lymphoma. There is no shared genetic ancestry found between the two groups. The molecular data that is available is extremely restricted, with only one instance reported, and lacking IgH rearrangement or somatic gene hypermutations. However, there is a distinction in cellular origins as cHL undergoes maturation within the germinal center whereas marginal and splenic MZLs develop outside the germinal center; both neoplasms are implicated in lymphomagenesis due to the activation of the NF-KB pathway [16]. Patients with composite cHL/DLBCL typically present with adenopathy but do not exhibit associated B symptoms, and they are older or middle-aged without a previous history of lymphoma, as in our presented case. Usually, the two groups are kept apart, with the cHL group composed of NS-subtype predominately and less often MC-subtype. CD15 and p53 are frequently detected in Hodgkin cells, but there have been no reports of TP53 mutation in cHL cases. Lymphomas with a sequential presentation have been found to have a less favorable prognosis compared to these lymphomas, which have been reported to have a better overall clinical outcome. A population-based study revealed that administering chemotherapy to patients with DLBCL resulted in a slight reduction in the percentage of relapse/death rate [17]. Singh and colleagues have recently published findings on six cases of sequential lymphomas involving either cHL/DLBCL or DLBCL/cHL; have found similar variants in both sets of lymphomas including TP53, TNFAIP3, B2M, and XPO1 which suggests a close genetic relationship between the two types of lymphomas and supports the concept of plasticity and common pathogenesis origin in mature B-cells [18]. Composite lymphomas involving cHL/NLPHL are rare occurrences among all composite lymphomas. The association between cHL and NLPHL, particularly in young males is given regardless of whether the presentation is synchronous or sequential. Despite the pathophysiological differences between cHL and NLPHL, there is evidence to suggest that having a family history of Hodgkin lymphoma predisposes individuals, particularly males, to a higher risk of developing the same condition. First-degree relatives of affected individuals have a cumulative risk of developing Hodgkin lymphoma of 0.6% [19]. Reported familial syndromes linked to Hodgkin lymphomas include conditions such as DICER1 syndrome, association with human leucocyte antigen (HLA) abnormalities, NPAT germinal mutation, germinal homozygote CD27 deficiency, KLHDC8B gene translocation, and familial KDR mutations associated syndrome [9]. Due to the lack of understanding regarding the precise development of this type of tumor, it is essential to undertake further research investigations to enhance our comprehension of lymphomagenesis. Composite cHL/T-cell lymphomas typically afflict individuals who are commonly aged in their middle years and elderly, and do not exhibit any previous signs of lymphoma at the time of diagnosis. The majority of the cHL group exhibits an MC-type. P53, CD30 and CD15 are frequently observed in Hodgkin cells. CD7 and CD4 are the T-cell markers that are expressed to a lesser extent in the T-cell lymphoma group. On the other hand, neoplastic T-cells consistently showed positivity for CD2 and CD3. Up until now, there is no molecular proof indicating a clonal connection between the two parts of these composite lymphomas. This is largely due to most molecular analyses being carried out on the whole sample, without separately examining the two parts. Furthermore, individualized contingents were observed in only two reported cases where no shared clonal gene for B or T gene-rearrangements were identified. Although cHL is commonly believed to originate from B-cells, certain cHL cases have been identified by some authors with T-cells clonal rearrangement of TCR-g and TCR-b in Hodgkin cells. Furthermore, there appears to be a shared clone of rearrangement in the TCR-encoding gene between both groups of patients with cHL/T-cell lymphomas [20]. In fact, cHL has been documented to occur in conjunction with other types of T-cell skin lymphomas and lymphoproliferative disorders, such as mycosis fungoides and lymphomatoid papulosis, regardless of the presence of TCR rearrangements status. The present review excluded these neoplasms because their pathologic development is likely to be different than the aim of our study. Lastly, someone must mention that in pathology practice, it could be highly deceptive to differentiate between peripheral T-cell lymphoma with Hodgkin-like cells from lymphocyte-rich type cHL or cHL/T-cell composite lymphoma. We believe it is likely that the occurrence of T-cell lymphoma with Hodgkin-like cells is more common than that of composite cHL/T-cell lymphoma. The cells resembling Hodgkin's lymphoma (RS-like cells) may or may not be EBV positive, particularly in T-cell lymphomas of T-follicular helper (TFH) origin. Their appearance is often attributed to the patient's weakened immune system, or a super imposed infection. If a pathologist encounters clusters of neoplastic T-cells with positive expression of TFH markers such as CD10, CXCL13 and OSCAR it should serve as a warning sign for T-cell lymphoma instead of cHL. It is ironic that other TFH-associated markers such as BCL6, PD1, and ICOS, are not as helpful because they can be found in the tumor milieu of cHL as well.

## Conclusions

In conclusion, it seems that cHL possesses a wider range of flexibility than what was previously believed. This can describe how it is linked to various B and T-cell lymphomas, as well as myeloid abnormalities. Composite lymphomas have a complex lymphomagenesis that is likely multifactorial, involving a combination of genetic, environmental, and infectious factors. We strongly recommend a tissue excisional biopsy for a more accurate assessment of pathology diagnosis, as the limited tissue material obtained through needle sampling may be insufficient or lead to incomplete pathology assessment.
